# {2,2′-[1,1′-(3-Aza­pentane-1,5-diyl­dinitrilo)diethyl­idyne]diphenolato}(piperidine)cobalt(III) tetra­phenyl­borate

**DOI:** 10.1107/S1600536808003875

**Published:** 2008-02-15

**Authors:** Soraia Meghdadi, Kurt J. Schenk, Mehdi Amirnasr, Farzaneh Fadaee

**Affiliations:** a Department of Chemistry, Isfahan University of Technology, Isfahan 84156-83111, Iran; b Laboratoire de Cristallographie, École Polytechnique Fédérale de Lausanne, Le Cubotron-521, Dorigny, CH-1015 Lausanne, Switzerland

## Abstract

The title compound, [Co(C_20_H_23_N_3_O_2_)(C_5_H_11_N)](C_24_H_20_B) or [Co{(Me-sal)_2_dien}(pprdn)]BPh_4_, where (Me-sal)_2_dien is 2,2′-[1,1′-(3-aza­pentane-1,5-diyldinitrilo)diethyl­idyne]diphenolate and pprdn is piperidine, contains a penta­dentate (Me-sal)_2_dien ligand furnishing an N_3_O_2_ set, such that two of the N and one of the O atoms of the salicyl­idene rings define three positions of an equatorial plane, whereas the secondary amine N atom and the other O atom of the salicyl­idene lie in axial positions. The piperidine ligand occupies an equatorial position *trans* to one of the imine N atoms of the salicyl­idene. In the observed conformation of the penta­dentate ligand, the salicyl­idene rings attain asymmetrical positions owing to the structural demands. The geometry of the resulting CoN_4_O_2_ coordination can be described as distorted octa­hedral. The asymetric unit contains two formula units.

## Related literature

For related literature, see: Amirnasr *et al.* (2001 ▶, 2006 ▶); Barnes *et al.* (1998 ▶); Botteher *et al.* (1997 ▶); Cini (2001 ▶); Hirota *et al.* (1998 ▶); Meghdadi *et al.* (2007 ▶); Morshedi *et al.* (2006 ▶); Munro & Govender (2007 ▶); Nagata *et al.* (1995 ▶); Niswander & Taylor (1997 ▶).
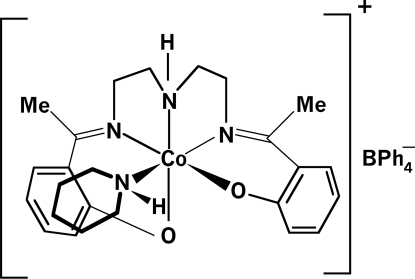


## Experimental

### 

#### Crystal data

[Co(C_20_H_23_N_3_O_2_)(C_5_H_11_N)](C_24_H_20_B)
*M* *_r_* = 800.70
Triclinic, 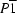

*a* = 11.1188 (7) Å
*b* = 16.8783 (10) Å
*c* = 23.3398 (13) Å
α = 91.222 (5)°
β = 96.054 (5)°
γ = 100.914 (5)°
*V* = 4273.1 (4) Å^3^
*Z* = 4
Mo *K*α radiation
μ = 0.45 mm^−1^
*T* = 293 (2) K
0.30 × 0.13 × 0.11 mm

#### Data collection

Stoe IPDSII diffractometer
Absorption correction: integration (*X-RED32*; Stoe & Cie, 2005 ▶) *T*_min_ = 0.878, *T*_max_ = 0.953
43249 measured reflections
22000 independent reflections
11758 reflections with *I* > 2σ(*I*)
*R*_int_ = 0.036

#### Refinement

*R*[*F* ^2^ > 2σ(*F* ^2^)] = 0.043
*wR*(*F* ^2^) = 0.119
*S* = 0.93
22000 reflections
1032 parameters
H-atom parameters constrained
Δρ_max_ = 0.33 e Å^−3^
Δρ_min_ = −0.41 e Å^−3^

### 

Data collection: *X-AREA* (Stoe & Cie, 2006 ▶); cell refinement: *X-AREA*; data reduction: *X-RED32* (Stoe & Cie, 2005 ▶); program(s) used to solve structure: *DIRDIF96* (Beurskens *et al.*, 1996 ▶); program(s) used to refine structure: *SHELXL97* (Sheldrick, 2008 ▶); molecular graphics: *SHELXTL* (Sheldrick, 2008 ▶) and *CrystalMaker* (Palmer, 2007 ▶); software used to prepare material for publication: *SHELXTL* and *PLATON* (Spek, 2003 ▶).

## Supplementary Material

Crystal structure: contains datablocks global, I. DOI: 10.1107/S1600536808003875/ci2550sup1.cif

Structure factors: contains datablocks I. DOI: 10.1107/S1600536808003875/ci2550Isup2.hkl

Additional supplementary materials:  crystallographic information; 3D view; checkCIF report

## Figures and Tables

**Table:** 

Co1—O11	1.8633 (14)
Co1—O21	1.8826 (14)
Co1—N21	1.9067 (18)
Co1—N11	1.9260 (18)
Co1—N3	1.9760 (18)
Co1—N4	2.0147 (17)

**Table:** 

O11—Co1—O21	87.66 (6)
O11—Co1—N21	95.77 (7)
O21—Co1—N21	87.14 (7)
O11—Co1—N11	91.80 (7)
O21—Co1—N11	179.46 (8)
N21—Co1—N11	92.89 (8)
O11—Co1—N3	176.26 (7)
O21—Co1—N3	96.03 (7)
N21—Co1—N3	85.04 (8)
N11—Co1—N3	84.51 (8)
O11—Co1—N4	84.45 (7)
O21—Co1—N4	83.94 (7)
N21—Co1—N4	171.06 (8)
N11—Co1—N4	96.03 (8)
N3—Co1—N4	95.32 (8)

**Table 2:** Hydrogen-bond geometry (Å, °) *Cg*1, *Cg*2 and *Cg*3 are the centroids of the C130–C135, C150–C155 and C230–C235 rings, respectively.

*D*—H⋯*A*	*D*—H	H⋯*A*	*D*⋯*A*	*D*—H⋯*A*
C45—H45*B*⋯O21	0.97	2.37	2.932 (3)	116
C85—H85*A*⋯O61	0.97	2.33	2.907 (3)	117
C135—H135⋯O21	0.93	2.58	3.289 (3)	133
C231—H231⋯O61	0.93	2.54	3.261 (3)	135
C18—H18*C*⋯*Cg*1^i^	0.96	2.98	3.559 (3)	120
C610—H61*A*⋯*Cg*1	0.97	2.94	3.886 (2)	165
C45—H45*A*⋯*Cg*2	0.97	3.00	3.658 (3)	126
C210—H21*B*⋯*Cg*3	0.97	2.98	3.898 (2)	158

Symmetry code: (i) 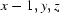.

**Fig. 1.**
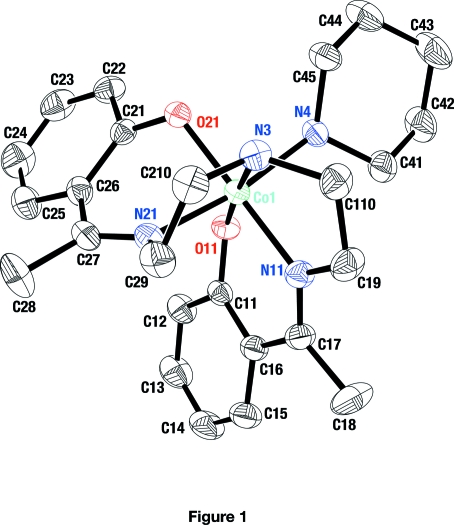
The structure of a independent [CoIII{(Me-sal)2dien}(pprdn)]+ cation. Displacement ellipsoids are drawn at the 30% probability level. The other independent cation and counter-ions have been omitted for clarity. H atoms are not shown.

**Fig. 2.**
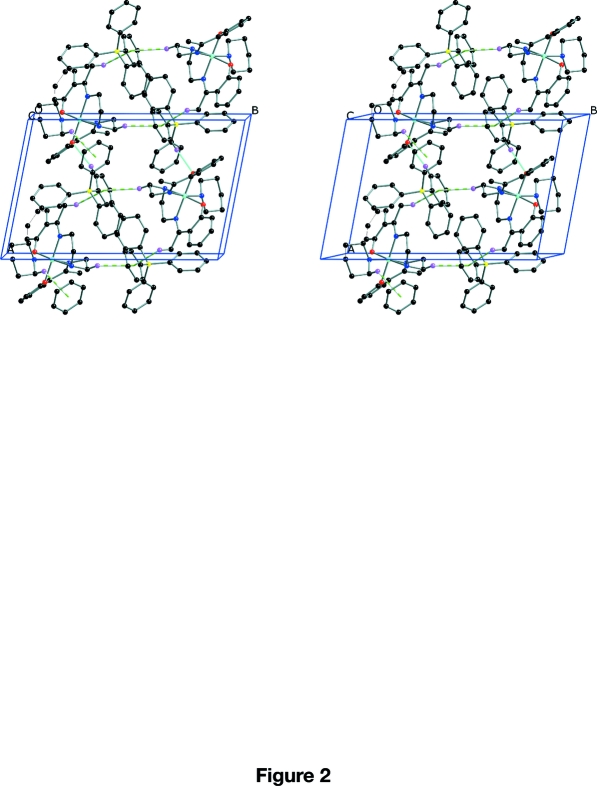
Stereoscopic view of the crystal structure of (I), illustrating some typical C–H···π interactions. H atoms not involved in the interactions have been omitted.

## supplementary crystallographic information

### Comment

Metal complexes of Schiff base ligands, aside from their wide applications, have been investigated from the coordination chemistry and structural points of view (Botteher *et al.*, 1997; Barnes *et al.*, 1998; Niswander & Taylor, 1997; Hirota *et al.*, 1998; Nagata *et al.*, 1995). Extensive investigation of the reactivity and structural aspects of metal complexes with pentadentate N_3_O_2_ ligands has been carried out and it has been found that, apart from the structural demands of the pentadentate ligand, many other factors play their rôles in the realised structures of these compounds (Cini, 2001; Amirnasr *et al.*, 2006; Morshedi *et al.*, 2006; Meghdadi *et al.*, 2007; Munro & Govender, 2007). In this context, we report here the synthesis and structure of [Co{(Me-sal)_2_dien}(pprdn)]BPh_4_, (I).

The asymmetric unit consists of two chemically identical, but conformationally slightly different [Co{(Me-sal)_2_dien}(pprdn)]^+^ cations and two tetraphenylborate anions. The environment surrounding each Co^III^ atom in (I) is distorted octahedral (Fig. 1) in which the three N atoms of the Schiff base ligand are arranged in facial positions. Selected geometric parameters are listed in Table 1; only the values of one complex are given, those of the other generally lie within 3σ level. The two chelate bite angles N3—Co1—N11/N21, formed by the two imine-N and the secondary amine-N of the Schiff base, are almost identical. The six-membered chelate rings formed by the phenolate-O and the imine-N atoms have, however, different bite angles O11—Co1—N11 and O21—Co1—N21. Of the three *trans* angles, only O21—Co1—N11 is close to ideal, but O11—Co1—N3 and N21—Co1—N4 deviate significantly. The Co—O(phenolate), Co—*N*(imine), Co—N(secondary amine) and Co—*N*(piperidine) bond lengths are comparable with those observed in related Co^III^ complexes (Meghdadi *et al.*, 2007, Amirnasr *et al.*, 2001). Neither of the two six-membered chelate rings formed by (Me-sal)_2_dien is planar. The metal centres lie off the salicylaldimine fragments by 0.666 (2), 1.040 (2), 0.730 (2) and 1.099 (2) Å for O11, O21, O51 and O61, respectively, the planes themselves having a mean deviation of 0.080 Å. The conformation adopted by (Me-sal)_2_dien in (I) is different from that of (Me-sal)_2_dpt in [Co{(Me-sal)_2_dpt}(py)_2_]PF_6_ (Meghdadi *et al.*, 2007). While the three donor N atoms of (Me-sal)_2_dpt occupy three meridional sites and the two phenolate-O atoms are *trans* to each other, the three N atoms of (Me-sal)_2_dien ligand in (I) are arranged in facial positions and the two phenolate-O atoms are *cis*. This is presumably due to the structural demands imparted by the (Me-sal)_2_dien Schiff base ligand which has forced the [Co{(Me-sal)_2_dien}(pprdn)]^+^ to attain such a twisted structure.

Unsurprisingly, the structure owes its cohesion to a multitude of C—H···π weak interactions (Fig. 2), the shortest of them (2.94 Å) being between the C610—H61A group and the C130—C135 phenyl ring (Table 2). There are also rather marginal C—H···O hydrogen bonds, some of them intramolecular.

### Experimental

To a stirring solution of Co(CH_3_COO)_2_.4H_2_O (0.249 g, 1 mmol) in methanol (25 ml) was added an equimolar amount of the H_2_(Me-sal)_2_dien ligand (0.339 g, 1 mmol). Owing to the formation of the [Co^II^{(Me-sal)_2_dien}] complex, the pink solution turned brown immediately. To this solution was then added piperidine (4 mmol) and air was bubbled through the reaction mixture for about 3 h. To the final green-brown solution was added NaBPh_4_ (0.342 g, 1 mmol). Dark green crystals of (I) suitable for X-ray analysis were obtained after 48 h. The crystals were filtered off, washed with cold methanol and dried under a vacuum. The measured crystal was bounded by the {010} and {001} pinacoids, the (101) pedion and a (701) cut face.

### Refinement

H atoms were positioned geometrically and allowed to ride on their parent atoms, with N—H = 0.91 Å and C—H = 0.93–0.97 Å. The *U*_iso_ values were constrained to be 1.5*U*_eq_ of the carrier atom for methyl H atoms and 1.2*U*_eq_ for the remaining H atoms. *PLATON* (Spek, 2003) detected solvent accessible voids of approximately 80 Å^3^ in the structure. These voids could have initially contained methanol solvent molecules but these molecules have since evaporated from the structure without degradation of the crystal.

### Crystal data

**Table:**

[Co(C_20_H_23_N_3_O_2_)(C_5_H_11_N)](C_24_H_20_B)	*Z* = 4
*M**_r_* = 800.70	*F*_000_ = 1696
Triclinic, *P*1	*D*_x_ = 1.245 Mg m^−^^3^
Hall symbol: -P 1	Mo *K*α radiation λ = 0.71073 Å
*a* = 11.1188 (7) Å	Cell parameters from 20476 reflections
*b* = 16.8783 (10) Å	θ = 1.8–29.6º
*c* = 23.3398 (13) Å	µ = 0.45 mm^−^^1^
α = 91.222 (5)º	*T* = 293 (2) K
β = 96.054 (5)º	Prism, green
γ = 100.914 (5)º	0.30 × 0.13 × 0.11 mm
*V* = 4273.1 (4) Å^3^	

### Data collection

**Table:**

Stoe IPDS2 diffractometer	22000 independent reflections
Monochromator: plane graphite	11758 reflections with *I* > 2σ(*I*)
Detector resolution: 6.67 pixels mm^-1^	*R*_int_ = 0.036
*T* = 293(2) K	θ_max_ = 29.6º
rotation scans	θ_min_ = 1.9º
Absorption correction: integration(X-RED32; Stoe & Cie, 2005)	*h* = −14→15
*T*_min_ = 0.878, *T*_max_ = 0.953	*k* = −23→23
43249 measured reflections	*l* = −32→28

### Refinement

**Table:**

Refinement on *F*^2^	Hydrogen site location: inferred from neighbouring sites
Least-squares matrix: full	H-atom parameters constrained
*R*[*F*^2^ > 2σ(*F*^2^)] = 0.043	*w* = 1/[σ^2^(*F*_o_^2^) + (0.0558*P*)^2^] where *P* = (*F*_o_^2^ + 2*F*_c_^2^)/3
*wR*(*F*^2^) = 0.119	(Δ/σ)_max_ = 0.027
*S* = 0.93	Δρ_max_ = 0.33 e Å^−^^3^
22000 reflections	Δρ_min_ = −0.41 e Å^−^^3^
1032 parameters	Extinction correction: SHELXL97 (Sheldrick, 2008), Fc^*^=kFc[1+0.001xFc^2^λ^3^/sin(2θ)]^-1/4^
Primary atom site location: structure-invariant direct methods	Extinction coefficient: 0.00168 (18)
Secondary atom site location: difference Fourier map	

### Special details

**Table:**

Geometry. All e.s.d.'s (except the e.s.d. in the dihedral angle between two l.s. planes) are estimated using the full covariance matrix. The cell e.s.d.'s are taken into account individually in the estimation of e.s.d.'s in distances, angles and torsion angles; correlations between e.s.d.'s in cell parameters are only used when they are defined by crystal symmetry. An approximate (isotropic) treatment of cell e.s.d.'s is used for estimating e.s.d.'s involving l.s. planes.
Refinement. Refinement of *F*^2^ against ALL reflections. The weighted *R*-factor *wR* and goodness of fit *S* are based on *F*^2^, conventional *R*-factors *R* are based on *F*, with *F* set to zero for negative *F*^2^. The threshold expression of *F*^2^ > σ(*F*^2^) is used only for calculating *R*-factors(gt) *etc*. and is not relevant to the choice of reflections for refinement. *R*-factors based on *F*^2^ are statistically about twice as large as those based on *F*, and *R*- factors based on ALL data will be even larger.

### Fractional atomic coordinates and isotropic or equivalent isotropic displacement parameters (Å^2^)

**Table:**

	*x*	*y*	*z*	*U*_iso_*/*U*_eq_	
Co1	0.03516 (2)	0.219640 (15)	0.197172 (12)	0.03749 (8)	
O11	−0.01627 (13)	0.12993 (8)	0.14649 (7)	0.0438 (3)	
O21	0.19796 (13)	0.20517 (8)	0.19338 (6)	0.0427 (3)	
N3	0.07829 (17)	0.31455 (10)	0.25161 (8)	0.0466 (4)	
H3	0.1475	0.3107	0.2751	0.056*	
N4	0.03413 (16)	0.13725 (10)	0.25841 (8)	0.0447 (4)	
H4	0.0572	0.0961	0.2392	0.054*	
N11	−0.13195 (17)	0.23359 (11)	0.20053 (8)	0.0466 (4)	
N21	0.06242 (17)	0.29650 (10)	0.13893 (8)	0.0465 (4)	
C11	−0.1080 (2)	0.12555 (12)	0.10570 (10)	0.0422 (5)	
C12	−0.1029 (3)	0.08125 (14)	0.05463 (11)	0.0573 (6)	
H12	−0.0341	0.0585	0.0506	0.069*	
C13	−0.1966 (3)	0.07100 (17)	0.01094 (13)	0.0723 (8)	
H13	−0.1910	0.0414	−0.0224	0.087*	
C14	−0.2998 (3)	0.10411 (19)	0.01560 (14)	0.0762 (8)	
H14	−0.3635	0.0968	−0.0144	0.091*	
C15	−0.3074 (2)	0.14765 (16)	0.06468 (13)	0.0629 (7)	
H15	−0.3772	0.1697	0.0676	0.075*	
C16	−0.2129 (2)	0.16020 (13)	0.11089 (10)	0.0475 (5)	
C17	−0.2249 (2)	0.20767 (14)	0.16245 (11)	0.0504 (5)	
C18	−0.3493 (2)	0.2266 (2)	0.17064 (16)	0.0852 (10)	
H18A	−0.3593	0.2276	0.2110	0.128*	
H18B	−0.4128	0.1859	0.1506	0.128*	
H18C	−0.3552	0.2783	0.1555	0.128*	
C19	−0.1470 (2)	0.28695 (16)	0.24924 (12)	0.0603 (7)	
H19B	−0.2091	0.2589	0.2718	0.072*	
H19A	−0.1743	0.3347	0.2346	0.072*	
C110	−0.0258 (2)	0.31131 (15)	0.28686 (11)	0.0573 (6)	
H11A	−0.0216	0.3639	0.3054	0.069*	
H11B	−0.0200	0.2726	0.3167	0.069*	
C21	0.22754 (19)	0.18656 (12)	0.14211 (10)	0.0428 (5)	
C22	0.3042 (2)	0.13029 (15)	0.13846 (12)	0.0563 (6)	
H22	0.3326	0.1069	0.1717	0.068*	
C23	0.3379 (3)	0.10928 (18)	0.08594 (15)	0.0726 (8)	
H23	0.3887	0.0716	0.0841	0.087*	
C24	0.2979 (3)	0.1430 (2)	0.03640 (15)	0.0811 (9)	
H24	0.3202	0.1274	0.0012	0.097*	
C25	0.2250 (3)	0.19957 (17)	0.03875 (12)	0.0670 (7)	
H25	0.1997	0.2231	0.0051	0.080*	
C26	0.1879 (2)	0.22264 (13)	0.09135 (10)	0.0490 (5)	
C27	0.1231 (2)	0.28984 (14)	0.09549 (11)	0.0518 (6)	
C28	0.1378 (4)	0.35382 (19)	0.05150 (14)	0.0873 (10)	
H28A	0.0613	0.3720	0.0431	0.131*	
H28B	0.1604	0.3318	0.0168	0.131*	
H28C	0.2010	0.3985	0.0665	0.131*	
C29	0.0356 (3)	0.37407 (13)	0.15872 (12)	0.0596 (7)	
H29A	−0.0522	0.3705	0.1597	0.072*	
H29B	0.0654	0.4168	0.1336	0.072*	
C210	0.1035 (2)	0.38914 (13)	0.21831 (12)	0.0572 (6)	
H21A	0.1913	0.4046	0.2158	0.069*	
H21B	0.0769	0.4330	0.2379	0.069*	
C41	−0.0832 (2)	0.09646 (16)	0.27676 (13)	0.0630 (7)	
H41A	−0.1428	0.0815	0.2430	0.076*	
H41B	−0.1142	0.1337	0.3010	0.076*	
C42	−0.0712 (3)	0.02150 (18)	0.30976 (14)	0.0778 (9)	
H42A	−0.1493	−0.0005	0.3236	0.093*	
H42B	−0.0515	−0.0190	0.2840	0.093*	
C43	0.0268 (3)	0.0396 (2)	0.35967 (15)	0.0901 (10)	
H43A	0.0368	−0.0102	0.3779	0.108*	
H43B	0.0023	0.0747	0.3879	0.108*	
C44	0.1480 (3)	0.08016 (17)	0.33991 (14)	0.0742 (8)	
H44B	0.2089	0.0951	0.3731	0.089*	
H44A	0.1773	0.0426	0.3152	0.089*	
C45	0.1328 (2)	0.15452 (15)	0.30733 (12)	0.0587 (6)	
H45A	0.1137	0.1945	0.3336	0.070*	
H45B	0.2100	0.1772	0.2929	0.070*	
Co2	0.57353 (3)	0.773334 (16)	0.298320 (13)	0.03930 (8)	
N7	0.52384 (17)	0.67418 (11)	0.24852 (9)	0.0492 (5)	
H7	0.4541	0.6774	0.2252	0.059*	
N8	0.57452 (17)	0.84923 (11)	0.23202 (8)	0.0473 (4)	
H8	0.5582	0.8940	0.2497	0.057*	
N51	0.73755 (17)	0.75432 (11)	0.29545 (9)	0.0480 (4)	
N61	0.54512 (17)	0.70276 (11)	0.36038 (9)	0.0489 (4)	
O61	0.41327 (13)	0.79323 (9)	0.30228 (7)	0.0448 (3)	
O51	0.63365 (14)	0.86782 (8)	0.34407 (7)	0.0452 (3)	
C51	0.7240 (2)	0.87176 (12)	0.38581 (10)	0.0419 (5)	
C52	0.7243 (3)	0.92242 (14)	0.43431 (11)	0.0555 (6)	
H52	0.6595	0.9496	0.4365	0.067*	
C53	0.8174 (3)	0.93252 (16)	0.47812 (12)	0.0659 (7)	
H53	0.8160	0.9670	0.5096	0.079*	
C54	0.9143 (3)	0.89204 (17)	0.47644 (12)	0.0658 (7)	
H54	0.9769	0.8984	0.5069	0.079*	
C55	0.9171 (2)	0.84263 (15)	0.42965 (12)	0.0580 (6)	
H55	0.9826	0.8158	0.4285	0.070*	
C56	0.8233 (2)	0.83143 (13)	0.38309 (10)	0.0461 (5)	
C57	0.8324 (2)	0.78085 (14)	0.33280 (11)	0.0509 (6)	
C58	0.9549 (2)	0.7592 (2)	0.32530 (16)	0.0855 (10)	
H58A	0.9642	0.7556	0.2850	0.128*	
H58B	0.9590	0.7081	0.3419	0.128*	
H58C	1.0197	0.8000	0.3443	0.128*	
C59	0.7488 (2)	0.69713 (17)	0.24871 (13)	0.0643 (7)	
H59A	0.7749	0.6499	0.2649	0.077*	
H59B	0.8104	0.7223	0.2248	0.077*	
C510	0.6256 (2)	0.67211 (15)	0.21238 (12)	0.0588 (6)	
H51A	0.6200	0.7085	0.1810	0.071*	
H51B	0.6185	0.6180	0.1959	0.071*	
C61	0.3910 (2)	0.81917 (15)	0.35295 (11)	0.0509 (6)	
C62	0.3228 (3)	0.88015 (19)	0.35462 (14)	0.0768 (9)	
H62	0.2935	0.9007	0.3204	0.092*	
C63	0.2986 (4)	0.9101 (3)	0.40639 (17)	0.1161 (16)	
H63	0.2528	0.9508	0.4067	0.139*	
C64	0.3405 (5)	0.8813 (3)	0.45736 (16)	0.1207 (16)	
H64	0.3258	0.9037	0.4921	0.145*	
C65	0.4042 (3)	0.8194 (2)	0.45728 (14)	0.0861 (10)	
H65	0.4304	0.7989	0.4921	0.103*	
C66	0.4304 (2)	0.78668 (16)	0.40525 (11)	0.0558 (6)	
C67	0.4856 (2)	0.71562 (15)	0.40341 (11)	0.0536 (6)	
C68	0.4633 (3)	0.65494 (19)	0.44956 (14)	0.0817 (9)	
H68A	0.5351	0.6316	0.4580	0.123*	
H68B	0.3940	0.6132	0.4362	0.123*	
H68C	0.4465	0.6815	0.4838	0.123*	
C69	0.5671 (3)	0.62214 (14)	0.34420 (13)	0.0634 (7)	
H69A	0.5358	0.5826	0.3712	0.076*	
H69B	0.6544	0.6232	0.3433	0.076*	
C610	0.4980 (2)	0.60292 (13)	0.28493 (13)	0.0612 (7)	
H61A	0.5232	0.5571	0.2672	0.073*	
H61B	0.4102	0.5888	0.2879	0.073*	
C81	0.6899 (2)	0.88312 (17)	0.20845 (13)	0.0676 (7)	
H81A	0.7136	0.8417	0.1850	0.081*	
H81B	0.7546	0.8994	0.2400	0.081*	
C82	0.6781 (3)	0.9555 (2)	0.17201 (16)	0.0869 (10)	
H82A	0.6665	0.9997	0.1967	0.104*	
H82B	0.7540	0.9727	0.1549	0.104*	
C83	0.5728 (3)	0.9370 (2)	0.12519 (15)	0.0898 (10)	
H83	0.5888	0.8977	0.0975	0.108*	
H83B	0.5642	0.9858	0.1052	0.108*	
C85	0.4690 (2)	0.83100 (16)	0.18587 (11)	0.0587 (6)	
H85A	0.3938	0.8122	0.2033	0.070*	
H85B	0.4813	0.7879	0.1603	0.070*	
C84	0.4541 (3)	0.90359 (18)	0.15098 (13)	0.0759 (8)	
H84A	0.4335	0.9450	0.1755	0.091*	
H84B	0.3872	0.8882	0.1203	0.091*	
B1	0.5386 (2)	0.33267 (14)	0.34545 (11)	0.0444 (6)	
C130	0.53836 (19)	0.36952 (12)	0.28052 (10)	0.0428 (5)	
C131	0.6387 (2)	0.42068 (13)	0.26134 (11)	0.0478 (5)	
H131	0.7077	0.4394	0.2876	0.057*	
C132	0.6403 (2)	0.44500 (14)	0.20490 (12)	0.0538 (6)	
H132	0.7102	0.4782	0.1941	0.065*	
C133	0.5399 (2)	0.42051 (14)	0.16509 (12)	0.0555 (6)	
H133	0.5409	0.4365	0.1272	0.067*	
C134	0.4374 (2)	0.37154 (14)	0.18237 (11)	0.0552 (6)	
H134	0.3678	0.3548	0.1560	0.066*	
C135	0.4372 (2)	0.34730 (14)	0.23833 (11)	0.0503 (5)	
H135	0.3663	0.3145	0.2487	0.060*	
C140	0.5641 (2)	0.23936 (13)	0.34129 (11)	0.0492 (5)	
C141	0.4984 (3)	0.18345 (15)	0.29914 (13)	0.0655 (7)	
H141	0.4437	0.2007	0.2715	0.079*	
C142	0.5108 (3)	0.10377 (17)	0.29648 (15)	0.0848 (10)	
H142	0.4629	0.0686	0.2680	0.102*	
C143	0.5922 (4)	0.07573 (18)	0.33499 (15)	0.0883 (11)	
H143	0.6015	0.0222	0.3327	0.106*	
C144	0.6597 (4)	0.1283 (2)	0.37702 (15)	0.0850 (10)	
H144	0.7160	0.1103	0.4036	0.102*	
C145	0.6453 (3)	0.20826 (17)	0.38056 (13)	0.0679 (7)	
H145	0.6913	0.2423	0.4101	0.081*	
C150	0.4045 (2)	0.32980 (13)	0.36988 (10)	0.0462 (5)	
C151	0.3600 (2)	0.27291 (15)	0.40849 (11)	0.0571 (6)	
H151	0.4033	0.2319	0.4172	0.069*	
C152	0.2538 (3)	0.27442 (19)	0.43471 (13)	0.0738 (8)	
H152	0.2284	0.2355	0.4608	0.089*	
C153	0.1868 (3)	0.3332 (2)	0.42213 (14)	0.0760 (9)	
H153	0.1150	0.3340	0.4391	0.091*	
C154	0.2264 (3)	0.39068 (18)	0.38422 (13)	0.0689 (8)	
H154	0.1820	0.4313	0.3758	0.083*	
C155	0.3325 (2)	0.38861 (15)	0.35834 (12)	0.0561 (6)	
H155	0.3568	0.4278	0.3323	0.067*	
C160	0.6443 (2)	0.39159 (13)	0.38997 (10)	0.0471 (5)	
C161	0.6175 (2)	0.44683 (13)	0.42957 (11)	0.0514 (6)	
H161	0.5353	0.4488	0.4324	0.062*	
C162	0.7073 (3)	0.49888 (16)	0.46492 (13)	0.0687 (7)	
H162	0.6849	0.5350	0.4906	0.082*	
C163	0.8295 (3)	0.49729 (19)	0.46222 (15)	0.0799 (9)	
H163	0.8902	0.5324	0.4858	0.096*	
C164	0.8613 (3)	0.4435 (2)	0.42447 (14)	0.0736 (8)	
H164	0.9438	0.4415	0.4224	0.088*	
C165	0.7701 (2)	0.39227 (17)	0.38956 (12)	0.0628 (7)	
H165	0.7936	0.3562	0.3643	0.075*	
B2	0.0681 (2)	0.66578 (15)	0.15211 (12)	0.0454 (6)	
C230	0.07126 (19)	0.63006 (12)	0.21744 (10)	0.0448 (5)	
C231	0.1725 (2)	0.65428 (14)	0.25919 (11)	0.0515 (6)	
H231	0.2412	0.6892	0.2488	0.062*	
C232	0.1752 (2)	0.62875 (15)	0.31501 (12)	0.0577 (6)	
H232	0.2445	0.6470	0.3412	0.069*	
C233	0.0756 (2)	0.57623 (15)	0.33230 (12)	0.0598 (6)	
H233	0.0765	0.5593	0.3700	0.072*	
C234	−0.0244 (2)	0.54981 (15)	0.29234 (12)	0.0584 (6)	
H234	−0.0920	0.5139	0.3029	0.070*	
C235	−0.0263 (2)	0.57595 (13)	0.23633 (11)	0.0503 (5)	
H235	−0.0954	0.5566	0.2103	0.060*	
C240	0.0555 (2)	0.76153 (13)	0.15400 (10)	0.0472 (5)	
C241	0.1118 (3)	0.81657 (15)	0.19836 (12)	0.0614 (7)	
H241	0.1542	0.7979	0.2302	0.074*	
C242	0.1076 (3)	0.89791 (17)	0.19723 (15)	0.0807 (9)	
H242	0.1481	0.9324	0.2278	0.097*	
C243	0.0452 (3)	0.92849 (16)	0.15219 (15)	0.0774 (9)	
H243	0.0409	0.9830	0.1521	0.093*	
C244	−0.0105 (3)	0.87740 (17)	0.10761 (15)	0.0812 (9)	
H244	−0.0531	0.8969	0.0762	0.097*	
C245	−0.0045 (3)	0.79625 (15)	0.10847 (13)	0.0687 (7)	
H245	−0.0426	0.7630	0.0769	0.082*	
C250	−0.0485 (2)	0.61028 (13)	0.11133 (10)	0.0442 (5)	
C251	−0.1693 (2)	0.62181 (16)	0.11131 (11)	0.0570 (6)	
H251	−0.1831	0.6661	0.1323	0.068*	
C253	−0.2536 (3)	0.50567 (17)	0.05002 (12)	0.0663 (7)	
H253	−0.3208	0.4711	0.0301	0.080*	
C254	−0.1370 (3)	0.49199 (15)	0.04799 (12)	0.0632 (7)	
H254	−0.1246	0.4477	0.0266	0.076*	
C252	−0.2700 (2)	0.57074 (18)	0.08164 (12)	0.0649 (7)	
H252	−0.3489	0.5809	0.0833	0.078*	
C255	−0.0374 (2)	0.54352 (13)	0.07755 (11)	0.0507 (6)	
H255	0.0410	0.5331	0.0748	0.061*	
C260	0.1965 (2)	0.65885 (13)	0.12433 (11)	0.0465 (5)	
C261	0.2385 (2)	0.70755 (15)	0.08037 (12)	0.0573 (6)	
H261	0.1957	0.7476	0.0683	0.069*	
C262	0.3409 (3)	0.69894 (18)	0.05384 (13)	0.0701 (8)	
H262	0.3650	0.7328	0.0245	0.084*	
C263	0.4067 (3)	0.64166 (19)	0.07011 (14)	0.0705 (8)	
H263	0.4761	0.6364	0.0524	0.085*	
C264	0.3694 (2)	0.59149 (17)	0.11305 (13)	0.0647 (7)	
H264	0.4130	0.5515	0.1243	0.078*	
C265	0.2663 (2)	0.60053 (14)	0.13974 (12)	0.0548 (6)	
H265	0.2430	0.5663	0.1690	0.066*	

### Atomic displacement parameters (Å^2^)

**Table:**

	*U*^11^	*U*^22^	*U*^33^	*U*^12^	*U*^13^	*U*^23^
Co1	0.03951 (15)	0.03579 (14)	0.03717 (18)	0.00805 (11)	0.00295 (13)	0.00127 (11)
O11	0.0473 (8)	0.0398 (7)	0.0430 (9)	0.0099 (6)	−0.0025 (7)	−0.0028 (6)
O21	0.0426 (8)	0.0457 (8)	0.0396 (9)	0.0090 (6)	0.0024 (7)	0.0019 (6)
N3	0.0491 (10)	0.0432 (9)	0.0474 (12)	0.0101 (8)	0.0042 (9)	−0.0059 (8)
N4	0.0501 (10)	0.0430 (9)	0.0386 (11)	0.0042 (7)	0.0019 (8)	0.0059 (7)
N11	0.0462 (10)	0.0502 (10)	0.0440 (12)	0.0128 (8)	0.0022 (9)	−0.0016 (8)
N21	0.0539 (11)	0.0406 (9)	0.0458 (12)	0.0129 (8)	0.0026 (9)	0.0045 (8)
C11	0.0478 (11)	0.0375 (10)	0.0382 (13)	0.0021 (8)	0.0006 (10)	0.0046 (8)
C12	0.0701 (16)	0.0525 (13)	0.0461 (16)	0.0072 (11)	0.0016 (13)	−0.0043 (10)
C13	0.091 (2)	0.0711 (18)	0.0451 (18)	0.0012 (15)	−0.0083 (16)	−0.0112 (13)
C14	0.0749 (19)	0.090 (2)	0.051 (2)	0.0014 (16)	−0.0216 (15)	0.0019 (15)
C15	0.0559 (14)	0.0695 (16)	0.0586 (19)	0.0082 (12)	−0.0095 (13)	0.0121 (13)
C16	0.0456 (12)	0.0483 (12)	0.0451 (15)	0.0043 (9)	−0.0025 (10)	0.0070 (10)
C17	0.0417 (12)	0.0544 (13)	0.0541 (16)	0.0102 (10)	−0.0007 (11)	0.0045 (11)
C18	0.0450 (14)	0.113 (2)	0.099 (3)	0.0262 (15)	−0.0029 (15)	−0.0208 (19)
C19	0.0514 (13)	0.0721 (16)	0.0612 (18)	0.0214 (12)	0.0105 (12)	−0.0136 (13)
C110	0.0592 (14)	0.0610 (14)	0.0535 (16)	0.0145 (11)	0.0129 (12)	−0.0124 (11)
C21	0.0383 (10)	0.0426 (11)	0.0447 (14)	0.0025 (8)	0.0032 (10)	−0.0043 (9)
C22	0.0504 (13)	0.0581 (14)	0.0614 (17)	0.0163 (11)	0.0015 (12)	−0.0069 (11)
C23	0.0595 (16)	0.0798 (19)	0.082 (2)	0.0246 (14)	0.0113 (15)	−0.0201 (16)
C24	0.085 (2)	0.098 (2)	0.064 (2)	0.0240 (18)	0.0213 (18)	−0.0208 (17)
C25	0.0783 (18)	0.0778 (18)	0.0460 (17)	0.0156 (14)	0.0126 (14)	−0.0012 (12)
C26	0.0537 (13)	0.0511 (12)	0.0416 (14)	0.0069 (10)	0.0086 (11)	0.0011 (9)
C27	0.0619 (14)	0.0519 (13)	0.0405 (14)	0.0094 (10)	0.0024 (12)	0.0076 (10)
C28	0.134 (3)	0.0769 (19)	0.060 (2)	0.0308 (19)	0.028 (2)	0.0283 (15)
C29	0.0758 (16)	0.0393 (11)	0.0693 (19)	0.0224 (11)	0.0114 (14)	0.0111 (11)
C210	0.0668 (15)	0.0379 (11)	0.0677 (18)	0.0112 (10)	0.0103 (14)	−0.0056 (10)
C41	0.0588 (15)	0.0648 (15)	0.0593 (18)	−0.0048 (12)	0.0068 (13)	0.0128 (12)
C42	0.084 (2)	0.0671 (17)	0.072 (2)	−0.0125 (14)	0.0084 (17)	0.0231 (14)
C43	0.121 (3)	0.078 (2)	0.060 (2)	−0.0059 (18)	−0.005 (2)	0.0285 (15)
C44	0.095 (2)	0.0613 (16)	0.0581 (19)	0.0064 (14)	−0.0150 (16)	0.0126 (13)
C45	0.0630 (15)	0.0579 (14)	0.0496 (16)	0.0043 (11)	−0.0065 (13)	0.0078 (11)
Co2	0.04197 (16)	0.03797 (15)	0.03858 (18)	0.00919 (11)	0.00504 (13)	−0.00022 (11)
N7	0.0491 (10)	0.0443 (10)	0.0533 (13)	0.0097 (8)	0.0023 (9)	−0.0075 (8)
N8	0.0550 (11)	0.0465 (10)	0.0379 (11)	0.0035 (8)	0.0048 (9)	0.0021 (8)
N51	0.0458 (10)	0.0516 (10)	0.0471 (12)	0.0119 (8)	0.0052 (9)	−0.0082 (8)
N61	0.0529 (11)	0.0444 (10)	0.0512 (13)	0.0148 (8)	0.0027 (9)	0.0075 (8)
O61	0.0459 (8)	0.0521 (8)	0.0382 (9)	0.0135 (6)	0.0057 (7)	0.0036 (6)
O51	0.0520 (8)	0.0413 (8)	0.0432 (10)	0.0145 (6)	0.0011 (7)	−0.0028 (6)
C51	0.0490 (12)	0.0365 (10)	0.0385 (13)	0.0038 (8)	0.0040 (10)	0.0023 (8)
C52	0.0714 (16)	0.0490 (13)	0.0446 (15)	0.0113 (11)	0.0016 (13)	−0.0052 (10)
C53	0.086 (2)	0.0631 (16)	0.0433 (16)	0.0049 (14)	0.0033 (15)	−0.0109 (11)
C54	0.0702 (17)	0.0763 (18)	0.0431 (17)	0.0037 (14)	−0.0094 (13)	−0.0007 (12)
C55	0.0529 (13)	0.0639 (15)	0.0525 (17)	0.0040 (11)	−0.0020 (12)	0.0033 (12)
C56	0.0461 (11)	0.0466 (12)	0.0430 (14)	0.0035 (9)	0.0031 (10)	0.0009 (9)
C57	0.0434 (12)	0.0576 (13)	0.0518 (16)	0.0104 (10)	0.0061 (11)	−0.0044 (11)
C58	0.0480 (15)	0.113 (2)	0.096 (3)	0.0253 (15)	−0.0019 (15)	−0.0371 (19)
C59	0.0539 (14)	0.0749 (17)	0.0665 (19)	0.0213 (12)	0.0065 (13)	−0.0245 (13)
C510	0.0580 (14)	0.0609 (14)	0.0572 (17)	0.0133 (11)	0.0072 (12)	−0.0214 (12)
C61	0.0502 (13)	0.0622 (14)	0.0454 (15)	0.0195 (11)	0.0118 (11)	0.0073 (11)
C62	0.093 (2)	0.095 (2)	0.0591 (19)	0.0562 (18)	0.0153 (16)	0.0094 (15)
C63	0.163 (4)	0.149 (4)	0.073 (3)	0.110 (3)	0.033 (3)	0.004 (2)
C64	0.180 (4)	0.159 (4)	0.059 (2)	0.108 (3)	0.041 (3)	0.004 (2)
C65	0.110 (3)	0.119 (3)	0.0439 (18)	0.051 (2)	0.0218 (18)	0.0124 (16)
C66	0.0612 (14)	0.0675 (15)	0.0441 (15)	0.0203 (12)	0.0134 (12)	0.0119 (11)
C67	0.0563 (13)	0.0605 (14)	0.0434 (15)	0.0102 (11)	0.0025 (12)	0.0134 (11)
C68	0.102 (2)	0.084 (2)	0.063 (2)	0.0226 (17)	0.0164 (18)	0.0329 (16)
C69	0.0726 (16)	0.0427 (12)	0.078 (2)	0.0189 (11)	0.0082 (15)	0.0132 (12)
C610	0.0654 (15)	0.0371 (11)	0.080 (2)	0.0096 (10)	0.0069 (14)	−0.0031 (11)
C81	0.0586 (15)	0.0736 (17)	0.0650 (19)	−0.0049 (13)	0.0120 (14)	0.0136 (14)
C82	0.092 (2)	0.079 (2)	0.082 (2)	−0.0112 (17)	0.018 (2)	0.0253 (17)
C83	0.114 (3)	0.091 (2)	0.058 (2)	0.0042 (19)	0.006 (2)	0.0283 (16)
C85	0.0598 (14)	0.0682 (15)	0.0447 (16)	0.0056 (12)	0.0014 (12)	0.0071 (11)
C84	0.093 (2)	0.0795 (19)	0.0522 (19)	0.0147 (16)	−0.0044 (16)	0.0156 (14)
B1	0.0463 (13)	0.0435 (13)	0.0420 (15)	0.0056 (10)	0.0043 (12)	0.0007 (10)
C130	0.0432 (11)	0.0379 (10)	0.0467 (14)	0.0070 (8)	0.0040 (10)	0.0012 (9)
C131	0.0410 (11)	0.0442 (11)	0.0550 (15)	0.0024 (9)	0.0015 (10)	0.0029 (10)
C132	0.0507 (13)	0.0496 (12)	0.0598 (17)	0.0024 (10)	0.0120 (12)	0.0115 (11)
C133	0.0623 (15)	0.0547 (13)	0.0510 (16)	0.0135 (11)	0.0069 (13)	0.0137 (11)
C134	0.0529 (13)	0.0572 (14)	0.0504 (16)	0.0033 (10)	−0.0051 (12)	0.0058 (11)
C135	0.0454 (12)	0.0512 (12)	0.0509 (15)	0.0015 (9)	0.0036 (11)	0.0058 (10)
C140	0.0554 (13)	0.0489 (12)	0.0465 (15)	0.0136 (10)	0.0126 (11)	0.0072 (10)
C141	0.0805 (18)	0.0485 (14)	0.0657 (19)	0.0148 (12)	−0.0037 (15)	−0.0007 (12)
C142	0.122 (3)	0.0484 (15)	0.083 (2)	0.0233 (16)	−0.002 (2)	−0.0063 (14)
C143	0.144 (3)	0.0550 (16)	0.077 (2)	0.0437 (19)	0.019 (2)	0.0043 (15)
C144	0.118 (3)	0.079 (2)	0.071 (2)	0.0495 (19)	0.012 (2)	0.0223 (17)
C145	0.088 (2)	0.0655 (16)	0.0547 (18)	0.0292 (14)	0.0028 (15)	0.0073 (12)
C150	0.0469 (12)	0.0424 (11)	0.0457 (14)	0.0021 (9)	0.0026 (10)	−0.0053 (9)
C151	0.0589 (14)	0.0566 (14)	0.0524 (16)	0.0003 (11)	0.0105 (12)	0.0023 (11)
C152	0.0717 (18)	0.0779 (19)	0.066 (2)	−0.0100 (15)	0.0259 (16)	−0.0026 (14)
C153	0.0546 (16)	0.096 (2)	0.074 (2)	0.0022 (15)	0.0201 (15)	−0.0285 (17)
C154	0.0573 (15)	0.0782 (18)	0.072 (2)	0.0213 (13)	0.0011 (14)	−0.0235 (15)
C155	0.0554 (14)	0.0559 (14)	0.0558 (17)	0.0098 (11)	0.0050 (12)	−0.0062 (11)
C160	0.0468 (12)	0.0512 (12)	0.0421 (14)	0.0063 (9)	0.0037 (10)	0.0070 (9)
C161	0.0561 (13)	0.0463 (12)	0.0496 (15)	0.0079 (10)	−0.0005 (11)	0.0022 (10)
C162	0.0806 (19)	0.0596 (15)	0.0589 (19)	0.0075 (13)	−0.0118 (15)	−0.0095 (12)
C163	0.0692 (19)	0.084 (2)	0.072 (2)	−0.0053 (15)	−0.0217 (16)	−0.0062 (16)
C164	0.0479 (14)	0.101 (2)	0.064 (2)	0.0022 (14)	−0.0062 (14)	0.0020 (16)
C165	0.0507 (14)	0.0830 (18)	0.0525 (17)	0.0099 (12)	0.0015 (12)	−0.0016 (13)
B2	0.0422 (12)	0.0431 (13)	0.0511 (17)	0.0097 (10)	0.0028 (12)	0.0024 (11)
C230	0.0408 (11)	0.0406 (11)	0.0533 (15)	0.0102 (8)	0.0023 (10)	0.0023 (9)
C231	0.0420 (11)	0.0534 (13)	0.0574 (16)	0.0063 (9)	0.0010 (11)	0.0079 (11)
C232	0.0497 (13)	0.0651 (15)	0.0568 (18)	0.0136 (11)	−0.0066 (12)	0.0064 (12)
C233	0.0641 (16)	0.0625 (15)	0.0547 (17)	0.0172 (12)	0.0041 (13)	0.0143 (12)
C234	0.0552 (14)	0.0524 (13)	0.0683 (19)	0.0073 (11)	0.0127 (13)	0.0125 (12)
C235	0.0458 (12)	0.0440 (11)	0.0592 (16)	0.0062 (9)	0.0014 (11)	0.0034 (10)
C240	0.0501 (12)	0.0475 (12)	0.0469 (14)	0.0152 (9)	0.0077 (11)	0.0009 (10)
C241	0.0737 (17)	0.0538 (14)	0.0575 (18)	0.0205 (12)	−0.0036 (14)	−0.0005 (11)
C242	0.114 (3)	0.0530 (15)	0.073 (2)	0.0236 (16)	−0.0107 (19)	−0.0131 (14)
C243	0.111 (2)	0.0451 (14)	0.080 (2)	0.0285 (15)	0.0046 (19)	0.0001 (13)
C244	0.116 (3)	0.0588 (16)	0.073 (2)	0.0396 (16)	−0.0122 (19)	0.0053 (14)
C245	0.098 (2)	0.0522 (14)	0.0556 (18)	0.0277 (14)	−0.0144 (15)	−0.0019 (12)
C250	0.0440 (11)	0.0475 (11)	0.0422 (13)	0.0107 (9)	0.0055 (10)	0.0060 (9)
C251	0.0483 (13)	0.0729 (16)	0.0501 (16)	0.0146 (11)	0.0032 (12)	−0.0049 (12)
C253	0.0597 (16)	0.0787 (18)	0.0499 (17)	−0.0097 (13)	−0.0008 (13)	0.0024 (13)
C254	0.0749 (18)	0.0518 (14)	0.0575 (18)	−0.0004 (12)	0.0060 (14)	−0.0022 (11)
C252	0.0440 (13)	0.097 (2)	0.0515 (17)	0.0085 (13)	0.0057 (12)	0.0048 (14)
C255	0.0533 (13)	0.0453 (12)	0.0540 (16)	0.0098 (10)	0.0066 (11)	0.0050 (10)
C260	0.0430 (11)	0.0429 (11)	0.0522 (15)	0.0080 (9)	−0.0001 (10)	−0.0018 (9)
C261	0.0563 (14)	0.0589 (14)	0.0599 (17)	0.0168 (11)	0.0097 (13)	0.0076 (12)
C262	0.0657 (17)	0.0814 (19)	0.066 (2)	0.0152 (14)	0.0195 (15)	0.0079 (14)
C263	0.0532 (15)	0.090 (2)	0.072 (2)	0.0224 (14)	0.0122 (14)	−0.0105 (16)
C264	0.0569 (15)	0.0682 (16)	0.073 (2)	0.0291 (13)	−0.0029 (14)	−0.0090 (14)
C265	0.0518 (13)	0.0515 (13)	0.0625 (17)	0.0157 (10)	0.0031 (12)	0.0022 (11)

### Geometric parameters (Å, °)

**Table:**

Co1—O11	1.8633 (14)	C66—C67	1.449 (3)
Co1—O21	1.8826 (14)	C67—C68	1.509 (3)
Co1—N21	1.9067 (18)	C68—H68A	0.96
Co1—N11	1.9260 (18)	C68—H68B	0.96
Co1—N3	1.9760 (18)	C68—H68C	0.96
Co1—N4	2.0147 (17)	C69—C610	1.507 (4)
O11—C11	1.310 (3)	C69—H69A	0.97
O21—C21	1.321 (3)	C69—H69B	0.97
N3—C110	1.482 (3)	C610—H61A	0.97
N3—C210	1.490 (3)	C610—H61B	0.97
N3—H3	0.91	C81—C82	1.521 (4)
N4—C41	1.466 (3)	C81—H81A	0.97
N4—C45	1.481 (3)	C81—H81B	0.97
N4—H4	0.91	C82—C83	1.497 (5)
N11—C17	1.291 (3)	C82—H82A	0.97
N11—C19	1.478 (3)	C82—H82B	0.97
N21—C27	1.290 (3)	C83—C84	1.523 (4)
N21—C29	1.472 (3)	C83—H83	0.97
C11—C12	1.406 (3)	C83—H83B	0.97
C11—C16	1.415 (3)	C85—C84	1.511 (4)
C12—C13	1.363 (4)	C85—H85A	0.97
C12—H12	0.93	C85—H85B	0.97
C13—C14	1.381 (4)	C84—H84A	0.97
C13—H13	0.93	C84—H84B	0.97
C14—C15	1.367 (4)	B1—C150	1.645 (3)
C14—H14	0.93	B1—C160	1.645 (3)
C15—C16	1.407 (3)	B1—C130	1.650 (3)
C15—H15	0.93	B1—C140	1.655 (3)
C16—C17	1.463 (3)	C130—C131	1.396 (3)
C17—C18	1.508 (3)	C130—C135	1.403 (3)
C18—H18A	0.96	C131—C132	1.389 (3)
C18—H18B	0.96	C131—H131	0.93
C18—H18C	0.96	C132—C133	1.367 (4)
C19—C110	1.511 (4)	C132—H132	0.93
C19—H19B	0.97	C133—C134	1.379 (3)
C19—H19A	0.97	C133—H133	0.93
C110—H11A	0.97	C134—C135	1.377 (3)
C110—H11B	0.97	C134—H134	0.93
C21—C22	1.398 (3)	C135—H135	0.93
C21—C26	1.411 (3)	C140—C141	1.393 (4)
C22—C23	1.377 (4)	C140—C145	1.399 (4)
C22—H22	0.93	C141—C142	1.378 (4)
C23—C24	1.369 (4)	C141—H141	0.93
C23—H23	0.93	C142—C143	1.364 (5)
C24—C25	1.368 (4)	C142—H142	0.93
C24—H24	0.93	C143—C144	1.367 (5)
C25—C26	1.406 (3)	C143—H143	0.93
C25—H25	0.93	C144—C145	1.391 (4)
C26—C27	1.461 (3)	C144—H144	0.93
C27—C28	1.504 (3)	C145—H145	0.93
C28—H28A	0.96	C150—C151	1.389 (3)
C28—H28B	0.96	C150—C155	1.402 (3)
C28—H28C	0.96	C151—C152	1.389 (4)
C29—C210	1.503 (4)	C151—H151	0.93
C29—H29A	0.97	C152—C153	1.368 (4)
C29—H29B	0.97	C152—H152	0.93
C210—H21A	0.97	C153—C154	1.370 (4)
C210—H21B	0.97	C153—H153	0.93
C41—C42	1.515 (4)	C154—C155	1.387 (3)
C41—H41A	0.97	C154—H154	0.93
C41—H41B	0.97	C155—H155	0.93
C42—C43	1.492 (4)	C160—C161	1.393 (3)
C42—H42A	0.97	C160—C165	1.397 (3)
C42—H42B	0.97	C161—C162	1.383 (4)
C43—C44	1.514 (4)	C161—H161	0.93
C43—H43A	0.97	C162—C163	1.372 (4)
C43—H43B	0.97	C162—H162	0.93
C44—C45	1.509 (3)	C163—C164	1.371 (4)
C44—H44B	0.97	C163—H163	0.93
C44—H44A	0.97	C164—C165	1.380 (4)
C45—H45A	0.97	C164—H164	0.93
C45—H45B	0.97	C165—H165	0.93
Co2—O51	1.8717 (15)	B2—C250	1.645 (3)
Co2—O61	1.8859 (15)	B2—C240	1.648 (3)
Co2—N61	1.9090 (19)	B2—C230	1.651 (4)
Co2—N51	1.9183 (18)	B2—C260	1.651 (3)
Co2—N7	1.9720 (18)	C230—C235	1.396 (3)
Co2—N8	2.0294 (18)	C230—C231	1.400 (3)
N7—C510	1.486 (3)	C231—C232	1.380 (3)
N7—C610	1.488 (3)	C231—H231	0.93
N7—H7	0.91	C232—C233	1.383 (3)
N8—C81	1.470 (3)	C232—H232	0.93
N8—C85	1.488 (3)	C233—C234	1.372 (4)
N8—H8	0.91	C233—H233	0.93
N51—C57	1.297 (3)	C234—C235	1.388 (4)
N51—C59	1.472 (3)	C234—H234	0.93
N61—C67	1.296 (3)	C235—H235	0.93
N61—C69	1.476 (3)	C240—C241	1.389 (3)
O61—C61	1.316 (3)	C240—C245	1.394 (3)
O51—C51	1.315 (3)	C241—C242	1.383 (4)
C51—C52	1.404 (3)	C241—H241	0.93
C51—C56	1.408 (3)	C242—C243	1.363 (4)
C52—C53	1.360 (4)	C242—H242	0.93
C52—H52	0.93	C243—C244	1.354 (4)
C53—C54	1.384 (4)	C243—H243	0.93
C53—H53	0.93	C244—C245	1.385 (4)
C54—C55	1.367 (4)	C244—H244	0.93
C54—H54	0.93	C245—H245	0.93
C55—C56	1.408 (3)	C250—C251	1.393 (3)
C55—H55	0.93	C250—C255	1.394 (3)
C56—C57	1.460 (3)	C251—C252	1.386 (4)
C57—C58	1.502 (3)	C251—H251	0.93
C58—H58A	0.96	C253—C252	1.361 (4)
C58—H58B	0.96	C253—C254	1.364 (4)
C58—H58C	0.96	C253—H253	0.93
C59—C510	1.514 (4)	C254—C255	1.381 (4)
C59—H59A	0.97	C254—H254	0.93
C59—H59B	0.97	C252—H252	0.93
C510—H51A	0.97	C255—H255	0.93
C510—H51B	0.97	C260—C261	1.392 (3)
C61—C62	1.391 (3)	C260—C265	1.395 (3)
C61—C66	1.410 (3)	C261—C262	1.382 (3)
C62—C63	1.372 (4)	C261—H261	0.93
C62—H62	0.93	C262—C263	1.356 (4)
C63—C64	1.365 (5)	C262—H262	0.93
C63—H63	0.93	C263—C264	1.373 (4)
C64—C65	1.368 (5)	C263—H263	0.93
C64—H64	0.93	C264—C265	1.392 (3)
C65—C66	1.404 (4)	C264—H264	0.93
C65—H65	0.93	C265—H265	0.93
			
O11—Co1—O21	87.66 (6)	C63—C62—H62	119.8
O11—Co1—N21	95.77 (7)	C61—C62—H62	119.8
O21—Co1—N21	87.14 (7)	C64—C63—C62	121.2 (3)
O11—Co1—N11	91.80 (7)	C64—C63—H63	119.4
O21—Co1—N11	179.46 (8)	C62—C63—H63	119.4
N21—Co1—N11	92.89 (8)	C63—C64—C65	119.9 (3)
O11—Co1—N3	176.26 (7)	C63—C64—H64	120.1
O21—Co1—N3	96.03 (7)	C65—C64—H64	120.1
N21—Co1—N3	85.04 (8)	C64—C65—C66	120.7 (3)
N11—Co1—N3	84.51 (8)	C64—C65—H65	119.6
O11—Co1—N4	84.45 (7)	C66—C65—H65	119.6
O21—Co1—N4	83.94 (7)	C65—C66—C61	118.9 (2)
N21—Co1—N4	171.06 (8)	C65—C66—C67	121.8 (2)
N11—Co1—N4	96.03 (8)	C61—C66—C67	119.0 (2)
N3—Co1—N4	95.32 (8)	N61—C67—C66	119.6 (2)
C11—O11—Co1	122.28 (13)	N61—C67—C68	121.6 (2)
C21—O21—Co1	116.91 (14)	C66—C67—C68	118.7 (2)
C110—N3—C210	113.92 (18)	C67—C68—H68A	109.5
C110—N3—Co1	106.78 (13)	C67—C68—H68B	109.5
C210—N3—Co1	108.99 (15)	H68A—C68—H68B	109.5
C110—N3—H3	109.0	C67—C68—H68C	109.5
C210—N3—H3	109.0	H68A—C68—H68C	109.5
Co1—N3—H3	109.0	H68B—C68—H68C	109.5
C41—N4—C45	111.79 (19)	N61—C69—C610	104.45 (19)
C41—N4—Co1	119.86 (15)	N61—C69—H69A	110.9
C45—N4—Co1	117.22 (13)	C610—C69—H69A	110.9
C41—N4—H4	101.2	N61—C69—H69B	110.9
C45—N4—H4	101.2	C610—C69—H69B	110.9
Co1—N4—H4	101.2	H69A—C69—H69B	108.9
C17—N11—C19	119.27 (19)	N7—C610—C69	109.80 (19)
C17—N11—Co1	126.48 (16)	N7—C610—H61A	109.7
C19—N11—Co1	113.91 (15)	C69—C610—H61A	109.7
C27—N21—C29	122.67 (19)	N7—C610—H61B	109.7
C27—N21—Co1	125.59 (15)	C69—C610—H61B	109.7
C29—N21—Co1	109.70 (15)	H61A—C610—H61B	108.2
O11—C11—C12	117.4 (2)	N8—C81—C82	112.3 (2)
O11—C11—C16	124.0 (2)	N8—C81—H81A	109.1
C12—C11—C16	118.6 (2)	C82—C81—H81A	109.1
C13—C12—C11	121.2 (3)	N8—C81—H81B	109.1
C13—C12—H12	119.4	C82—C81—H81B	109.1
C11—C12—H12	119.4	H81A—C81—H81B	107.9
C12—C13—C14	120.8 (3)	C83—C82—C81	112.4 (3)
C12—C13—H13	119.6	C83—C82—H82A	109.1
C14—C13—H13	119.6	C81—C82—H82A	109.1
C15—C14—C13	119.3 (3)	C83—C82—H82B	109.1
C15—C14—H14	120.3	C81—C82—H82B	109.1
C13—C14—H14	120.3	H82A—C82—H82B	107.8
C14—C15—C16	122.1 (3)	C82—C83—C84	109.8 (3)
C14—C15—H15	119.0	C82—C83—H83	109.7
C16—C15—H15	119.0	C84—C83—H83	109.7
C15—C16—C11	118.0 (2)	C82—C83—H83B	109.7
C15—C16—C17	120.1 (2)	C84—C83—H83B	109.7
C11—C16—C17	121.9 (2)	H83—C83—H83B	108.2
N11—C17—C16	121.7 (2)	N8—C85—C84	112.5 (2)
N11—C17—C18	119.8 (2)	N8—C85—H85A	109.1
C16—C17—C18	118.6 (2)	C84—C85—H85A	109.1
C17—C18—H18A	109.5	N8—C85—H85B	109.1
C17—C18—H18B	109.5	C84—C85—H85B	109.1
H18A—C18—H18B	109.5	H85A—C85—H85B	107.8
C17—C18—H18C	109.5	C85—C84—C83	110.4 (3)
H18A—C18—H18C	109.5	C85—C84—H84A	109.6
H18B—C18—H18C	109.5	C83—C84—H84A	109.6
N11—C19—C110	109.70 (18)	C85—C84—H84B	109.6
N11—C19—H19B	109.7	C83—C84—H84B	109.6
C110—C19—H19B	109.7	H84A—C84—H84B	108.1
N11—C19—H19A	109.7	C150—B1—C160	107.98 (18)
C110—C19—H19A	109.7	C150—B1—C130	110.75 (19)
H19B—C19—H19A	108.2	C160—B1—C130	109.16 (17)
N3—C110—C19	110.2 (2)	C150—B1—C140	108.36 (17)
N3—C110—H11A	109.6	C160—B1—C140	111.98 (19)
C19—C110—H11A	109.6	C130—B1—C140	108.62 (18)
N3—C110—H11B	109.6	C131—C130—C135	114.0 (2)
C19—C110—H11B	109.6	C131—C130—B1	124.2 (2)
H11A—C110—H11B	108.1	C135—C130—B1	121.67 (18)
O21—C21—C22	118.3 (2)	C132—C131—C130	123.1 (2)
O21—C21—C26	122.83 (19)	C132—C131—H131	118.5
C22—C21—C26	118.8 (2)	C130—C131—H131	118.5
C23—C22—C21	120.4 (3)	C133—C132—C131	120.6 (2)
C23—C22—H22	119.8	C133—C132—H132	119.7
C21—C22—H22	119.8	C131—C132—H132	119.7
C24—C23—C22	121.0 (3)	C132—C133—C134	118.5 (2)
C24—C23—H23	119.5	C132—C133—H133	120.8
C22—C23—H23	119.5	C134—C133—H133	120.8
C25—C24—C23	120.0 (3)	C135—C134—C133	120.5 (2)
C25—C24—H24	120.0	C135—C134—H134	119.8
C23—C24—H24	120.0	C133—C134—H134	119.8
C24—C25—C26	121.0 (3)	C134—C135—C130	123.3 (2)
C24—C25—H25	119.5	C134—C135—H135	118.3
C26—C25—H25	119.5	C130—C135—H135	118.3
C25—C26—C21	118.8 (2)	C141—C140—C145	114.4 (2)
C25—C26—C27	121.3 (2)	C141—C140—B1	121.2 (2)
C21—C26—C27	119.4 (2)	C145—C140—B1	124.3 (2)
N21—C27—C26	119.6 (2)	C142—C141—C140	123.1 (3)
N21—C27—C28	121.3 (2)	C142—C141—H141	118.4
C26—C27—C28	118.9 (2)	C140—C141—H141	118.4
C27—C28—H28A	109.5	C143—C142—C141	120.9 (3)
C27—C28—H28B	109.5	C143—C142—H142	119.5
H28A—C28—H28B	109.5	C141—C142—H142	119.5
C27—C28—H28C	109.5	C142—C143—C144	118.4 (3)
H28A—C28—H28C	109.5	C142—C143—H143	120.8
H28B—C28—H28C	109.5	C144—C143—H143	120.8
N21—C29—C210	104.22 (18)	C143—C144—C145	120.8 (3)
N21—C29—H29A	110.9	C143—C144—H144	119.6
C210—C29—H29A	110.9	C145—C144—H144	119.6
N21—C29—H29B	110.9	C144—C145—C140	122.4 (3)
C210—C29—H29B	110.9	C144—C145—H145	118.8
H29A—C29—H29B	108.9	C140—C145—H145	118.8
N3—C210—C29	109.71 (19)	C151—C150—C155	114.7 (2)
N3—C210—H21A	109.7	C151—C150—B1	121.9 (2)
C29—C210—H21A	109.7	C155—C150—B1	123.1 (2)
N3—C210—H21B	109.7	C150—C151—C152	123.1 (3)
C29—C210—H21B	109.7	C150—C151—H151	118.4
H21A—C210—H21B	108.2	C152—C151—H151	118.4
N4—C41—C42	112.6 (2)	C153—C152—C151	120.0 (3)
N4—C41—H41A	109.1	C153—C152—H152	120.0
C42—C41—H41A	109.1	C151—C152—H152	120.0
N4—C41—H41B	109.1	C152—C153—C154	119.3 (3)
C42—C41—H41B	109.1	C152—C153—H153	120.4
H41A—C41—H41B	107.8	C154—C153—H153	120.4
C43—C42—C41	111.6 (2)	C153—C154—C155	120.2 (3)
C43—C42—H42A	109.3	C153—C154—H154	119.9
C41—C42—H42A	109.3	C155—C154—H154	119.9
C43—C42—H42B	109.3	C154—C155—C150	122.6 (3)
C41—C42—H42B	109.3	C154—C155—H155	118.7
H42A—C42—H42B	108.0	C150—C155—H155	118.7
C42—C43—C44	110.6 (3)	C161—C160—C165	114.2 (2)
C42—C43—H43A	109.5	C161—C160—B1	123.5 (2)
C44—C43—H43A	109.5	C165—C160—B1	122.3 (2)
C42—C43—H43B	109.5	C162—C161—C160	123.1 (2)
C44—C43—H43B	109.5	C162—C161—H161	118.5
H43A—C43—H43B	108.1	C160—C161—H161	118.5
C45—C44—C43	110.7 (3)	C163—C162—C161	120.1 (3)
C45—C44—H44B	109.5	C163—C162—H162	120.0
C43—C44—H44B	109.5	C161—C162—H162	120.0
C45—C44—H44A	109.5	C164—C163—C162	119.4 (3)
C43—C44—H44A	109.5	C164—C163—H163	120.3
H44B—C44—H44A	108.1	C162—C163—H163	120.3
N4—C45—C44	112.6 (2)	C163—C164—C165	119.5 (3)
N4—C45—H45A	109.1	C163—C164—H164	120.2
C44—C45—H45A	109.1	C165—C164—H164	120.2
N4—C45—H45B	109.1	C164—C165—C160	123.7 (3)
C44—C45—H45B	109.1	C164—C165—H165	118.1
H45A—C45—H45B	107.8	C160—C165—H165	118.1
O51—Co2—O61	88.02 (6)	C250—B2—C240	111.11 (19)
O51—Co2—N61	96.54 (8)	C250—B2—C230	108.09 (18)
O61—Co2—N61	87.10 (7)	C240—B2—C230	110.83 (19)
O51—Co2—N51	91.05 (7)	C250—B2—C260	108.47 (18)
O61—Co2—N51	179.01 (8)	C240—B2—C260	108.24 (18)
N61—Co2—N51	92.68 (8)	C230—B2—C260	110.08 (18)
O51—Co2—N7	175.38 (7)	C235—C230—C231	114.5 (2)
O61—Co2—N7	96.42 (7)	C235—C230—B2	123.6 (2)
N61—Co2—N7	85.01 (8)	C231—C230—B2	121.82 (19)
N51—Co2—N7	84.52 (8)	C232—C231—C230	123.1 (2)
O51—Co2—N8	84.50 (7)	C232—C231—H231	118.4
O61—Co2—N8	83.75 (7)	C230—C231—H231	118.4
N61—Co2—N8	170.75 (8)	C231—C232—C233	120.5 (2)
N51—Co2—N8	96.49 (8)	C231—C232—H232	119.7
N7—Co2—N8	94.67 (8)	C233—C232—H232	119.7
C510—N7—C610	113.80 (19)	C234—C233—C232	118.2 (2)
C510—N7—Co2	106.39 (13)	C234—C233—H233	120.9
C610—N7—Co2	109.53 (16)	C232—C233—H233	120.9
C510—N7—H7	109.0	C233—C234—C235	120.9 (2)
C610—N7—H7	109.0	C233—C234—H234	119.6
Co2—N7—H7	109.0	C235—C234—H234	119.6
C81—N8—C85	111.46 (19)	C234—C235—C230	122.8 (2)
C81—N8—Co2	120.86 (16)	C234—C235—H235	118.6
C85—N8—Co2	117.06 (14)	C230—C235—H235	118.6
C81—N8—H8	101.0	C241—C240—C245	113.6 (2)
C85—N8—H8	101.0	C241—C240—B2	123.6 (2)
Co2—N8—H8	101.0	C245—C240—B2	122.6 (2)
C57—N51—C59	119.18 (19)	C242—C241—C240	122.8 (3)
C57—N51—Co2	126.41 (15)	C242—C241—H241	118.6
C59—N51—Co2	114.11 (15)	C240—C241—H241	118.6
C67—N61—C69	122.9 (2)	C243—C242—C241	121.2 (3)
C67—N61—Co2	125.09 (16)	C243—C242—H242	119.4
C69—N61—Co2	109.77 (16)	C241—C242—H242	119.4
C61—O61—Co2	115.70 (15)	C244—C243—C242	118.2 (3)
C51—O51—Co2	121.60 (12)	C244—C243—H243	120.9
O51—C51—C52	117.8 (2)	C242—C243—H243	120.9
O51—C51—C56	123.63 (19)	C243—C244—C245	120.4 (3)
C52—C51—C56	118.5 (2)	C243—C244—H244	119.8
C53—C52—C51	121.3 (2)	C245—C244—H244	119.8
C53—C52—H52	119.4	C244—C245—C240	123.7 (3)
C51—C52—H52	119.4	C244—C245—H245	118.1
C52—C53—C54	120.7 (2)	C240—C245—H245	118.1
C52—C53—H53	119.6	C251—C250—C255	113.8 (2)
C54—C53—H53	119.6	C251—C250—B2	122.7 (2)
C55—C54—C53	119.4 (3)	C255—C250—B2	123.3 (2)
C55—C54—H54	120.3	C252—C251—C250	123.4 (2)
C53—C54—H54	120.3	C252—C251—H251	118.3
C54—C55—C56	121.6 (3)	C250—C251—H251	118.3
C54—C55—H55	119.2	C252—C253—C254	119.0 (3)
C56—C55—H55	119.2	C252—C253—H253	120.5
C55—C56—C51	118.5 (2)	C254—C253—H253	120.5
C55—C56—C57	119.9 (2)	C253—C254—C255	120.4 (3)
C51—C56—C57	121.6 (2)	C253—C254—H254	119.8
N51—C57—C56	121.5 (2)	C255—C254—H254	119.8
N51—C57—C58	120.2 (2)	C253—C252—C251	120.1 (3)
C56—C57—C58	118.3 (2)	C253—C252—H252	119.9
C57—C58—H58A	109.5	C251—C252—H252	119.9
C57—C58—H58B	109.5	C254—C255—C250	123.3 (2)
H58A—C58—H58B	109.5	C254—C255—H255	118.4
C57—C58—H58C	109.5	C250—C255—H255	118.4
H58A—C58—H58C	109.5	C261—C260—C265	114.7 (2)
H58B—C58—H58C	109.5	C261—C260—B2	122.0 (2)
N51—C59—C510	109.59 (18)	C265—C260—B2	123.1 (2)
N51—C59—H59A	109.8	C262—C261—C260	122.9 (2)
C510—C59—H59A	109.8	C262—C261—H261	118.6
N51—C59—H59B	109.8	C260—C261—H261	118.6
C510—C59—H59B	109.8	C263—C262—C261	120.7 (3)
H59A—C59—H59B	108.2	C263—C262—H262	119.6
N7—C510—C59	109.9 (2)	C261—C262—H262	119.6
N7—C510—H51A	109.7	C262—C263—C264	119.0 (2)
C59—C510—H51A	109.7	C262—C263—H263	120.5
N7—C510—H51B	109.7	C264—C263—H263	120.5
C59—C510—H51B	109.7	C263—C264—C265	120.0 (2)
H51A—C510—H51B	108.2	C263—C264—H264	120.0
O61—C61—C62	118.2 (2)	C265—C264—H264	120.0
O61—C61—C66	123.1 (2)	C264—C265—C260	122.6 (2)
C62—C61—C66	118.7 (2)	C264—C265—H265	118.7
C63—C62—C61	120.5 (3)	C260—C265—H265	118.7
			
O21—Co1—O11—C11	−142.51 (15)	Co2—N51—C57—C58	178.6 (2)
N21—Co1—O11—C11	−55.62 (16)	C55—C56—C57—N51	164.2 (2)
N11—Co1—O11—C11	37.47 (16)	C51—C56—C57—N51	−17.6 (3)
N4—Co1—O11—C11	133.36 (16)	C55—C56—C57—C58	−15.2 (4)
O11—Co1—O21—C21	41.59 (14)	C51—C56—C57—C58	163.0 (2)
N21—Co1—O21—C21	−54.31 (14)	C57—N51—C59—C510	177.5 (2)
N3—Co1—O21—C21	−139.01 (14)	Co2—N51—C59—C510	3.5 (3)
N4—Co1—O21—C21	126.25 (15)	C610—N7—C510—C59	−78.7 (3)
O21—Co1—N3—C110	−148.59 (15)	Co2—N7—C510—C59	42.0 (2)
N21—Co1—N3—C110	124.83 (16)	N51—C59—C510—N7	−30.1 (3)
N11—Co1—N3—C110	31.43 (16)	Co2—O61—C61—C62	138.9 (2)
N4—Co1—N3—C110	−64.14 (16)	Co2—O61—C61—C66	−42.7 (3)
O21—Co1—N3—C210	87.94 (15)	O61—C61—C62—C63	−179.0 (3)
N21—Co1—N3—C210	1.35 (15)	C66—C61—C62—C63	2.5 (5)
N11—Co1—N3—C210	−92.05 (15)	C61—C62—C63—C64	0.1 (7)
N4—Co1—N3—C210	172.38 (15)	C62—C63—C64—C65	−2.3 (8)
O11—Co1—N4—C41	−76.23 (18)	C63—C64—C65—C66	1.9 (7)
O21—Co1—N4—C41	−164.46 (18)	C64—C65—C66—C61	0.7 (5)
N11—Co1—N4—C41	15.00 (19)	C64—C65—C66—C67	−173.1 (4)
N3—Co1—N4—C41	100.02 (18)	O61—C61—C66—C65	178.8 (3)
O11—Co1—N4—C45	142.82 (17)	C62—C61—C66—C65	−2.8 (4)
O21—Co1—N4—C45	54.59 (17)	O61—C61—C66—C67	−7.3 (4)
N11—Co1—N4—C45	−125.95 (17)	C62—C61—C66—C67	171.1 (3)
N3—Co1—N4—C45	−40.93 (17)	C69—N61—C67—C66	−158.6 (2)
O11—Co1—N11—C17	−22.7 (2)	Co2—N61—C67—C66	2.6 (4)
N21—Co1—N11—C17	73.2 (2)	C69—N61—C67—C68	16.5 (4)
N3—Co1—N11—C17	157.9 (2)	Co2—N61—C67—C68	177.7 (2)
N4—Co1—N11—C17	−107.3 (2)	C65—C66—C67—N61	−157.0 (3)
O11—Co1—N11—C19	164.13 (17)	C61—C66—C67—N61	29.2 (4)
N21—Co1—N11—C19	−99.99 (18)	C65—C66—C67—C68	27.8 (4)
N3—Co1—N11—C19	−15.26 (17)	C61—C66—C67—C68	−145.9 (3)
N4—Co1—N11—C19	79.53 (18)	C67—N61—C69—C610	116.0 (3)
O11—Co1—N21—C27	−48.3 (2)	Co2—N61—C69—C610	−47.7 (2)
O21—Co1—N21—C27	39.0 (2)	C510—N7—C610—C69	94.6 (2)
N11—Co1—N21—C27	−140.4 (2)	Co2—N7—C610—C69	−24.3 (2)
N3—Co1—N21—C27	135.3 (2)	N61—C69—C610—N7	45.9 (3)
O11—Co1—N21—C29	147.64 (16)	C85—N8—C81—C82	52.9 (3)
O21—Co1—N21—C29	−125.00 (16)	Co2—N8—C81—C82	−163.5 (2)
N11—Co1—N21—C29	55.54 (16)	N8—C81—C82—C83	−54.0 (4)
N3—Co1—N21—C29	−28.69 (16)	C81—C82—C83—C84	54.4 (4)
Co1—O11—C11—C12	148.64 (16)	C81—N8—C85—C84	−55.1 (3)
Co1—O11—C11—C16	−33.6 (3)	Co2—N8—C85—C84	159.87 (19)
O11—C11—C12—C13	177.4 (2)	N8—C85—C84—C83	56.3 (3)
C16—C11—C12—C13	−0.5 (3)	C82—C83—C84—C85	−55.3 (4)
C11—C12—C13—C14	0.1 (4)	C150—B1—C130—C131	142.0 (2)
C12—C13—C14—C15	0.1 (5)	C160—B1—C130—C131	23.2 (3)
C13—C14—C15—C16	0.2 (4)	C140—B1—C130—C131	−99.2 (2)
C14—C15—C16—C11	−0.5 (4)	C150—B1—C130—C135	−42.0 (3)
C14—C15—C16—C17	179.6 (3)	C160—B1—C130—C135	−160.77 (19)
O11—C11—C16—C15	−177.1 (2)	C140—B1—C130—C135	76.9 (2)
C12—C11—C16—C15	0.7 (3)	C135—C130—C131—C132	−2.4 (3)
O11—C11—C16—C17	2.8 (3)	B1—C130—C131—C132	173.9 (2)
C12—C11—C16—C17	−179.5 (2)	C130—C131—C132—C133	1.4 (4)
C19—N11—C17—C16	174.9 (2)	C131—C132—C133—C134	0.3 (4)
Co1—N11—C17—C16	2.1 (3)	C132—C133—C134—C135	−0.8 (4)
C19—N11—C17—C18	−4.9 (4)	C133—C134—C135—C130	−0.4 (4)
Co1—N11—C17—C18	−177.7 (2)	C131—C130—C135—C134	1.9 (3)
C15—C16—C17—N11	−166.4 (2)	B1—C130—C135—C134	−174.5 (2)
C11—C16—C17—N11	13.8 (3)	C150—B1—C140—C141	72.0 (3)
C15—C16—C17—C18	13.4 (4)	C160—B1—C140—C141	−169.0 (2)
C11—C16—C17—C18	−166.4 (2)	C130—B1—C140—C141	−48.4 (3)
C17—N11—C19—C110	−178.3 (2)	C150—B1—C140—C145	−103.9 (3)
Co1—N11—C19—C110	−4.6 (3)	C160—B1—C140—C145	15.1 (3)
C210—N3—C110—C19	78.7 (2)	C130—B1—C140—C145	135.7 (2)
Co1—N3—C110—C19	−41.6 (2)	C145—C140—C141—C142	0.8 (4)
N11—C19—C110—N3	30.5 (3)	B1—C140—C141—C142	−175.5 (3)
Co1—O21—C21—C22	−140.95 (17)	C140—C141—C142—C143	−1.8 (5)
Co1—O21—C21—C26	40.8 (2)	C141—C142—C143—C144	1.2 (5)
O21—C21—C22—C23	−179.7 (2)	C142—C143—C144—C145	0.3 (5)
C26—C21—C22—C23	−1.5 (3)	C143—C144—C145—C140	−1.3 (5)
C21—C22—C23—C24	0.3 (4)	C141—C140—C145—C144	0.8 (4)
C22—C23—C24—C25	1.2 (5)	B1—C140—C145—C144	176.9 (3)
C23—C24—C25—C26	−1.5 (5)	C160—B1—C150—C151	−89.3 (3)
C24—C25—C26—C21	0.3 (4)	C130—B1—C150—C151	151.3 (2)
C24—C25—C26—C27	172.3 (3)	C140—B1—C150—C151	32.2 (3)
O21—C21—C26—C25	179.4 (2)	C160—B1—C150—C155	84.1 (3)
C22—C21—C26—C25	1.2 (3)	C130—B1—C150—C155	−35.4 (3)
O21—C21—C26—C27	7.1 (3)	C140—B1—C150—C155	−154.4 (2)
C22—C21—C26—C27	−171.1 (2)	C155—C150—C151—C152	−1.2 (4)
C29—N21—C27—C26	156.0 (2)	B1—C150—C151—C152	172.7 (2)
Co1—N21—C27—C26	−6.1 (3)	C150—C151—C152—C153	1.1 (4)
C29—N21—C27—C28	−18.7 (4)	C151—C152—C153—C154	−0.9 (5)
Co1—N21—C27—C28	179.2 (2)	C152—C153—C154—C155	0.9 (4)
C25—C26—C27—N21	161.9 (3)	C153—C154—C155—C150	−1.0 (4)
C21—C26—C27—N21	−26.1 (3)	C151—C150—C155—C154	1.1 (4)
C25—C26—C27—C28	−23.4 (4)	B1—C150—C155—C154	−172.7 (2)
C21—C26—C27—C28	148.7 (3)	C150—B1—C160—C161	−17.8 (3)
C27—N21—C29—C210	−116.2 (3)	C130—B1—C160—C161	102.7 (2)
Co1—N21—C29—C210	48.4 (2)	C140—B1—C160—C161	−137.0 (2)
C110—N3—C210—C29	−93.5 (2)	C150—B1—C160—C165	164.7 (2)
Co1—N3—C210—C29	25.6 (2)	C130—B1—C160—C165	−74.9 (3)
N21—C29—C210—N3	−47.2 (2)	C140—B1—C160—C165	45.5 (3)
C45—N4—C41—C42	−53.1 (3)	C165—C160—C161—C162	0.9 (4)
Co1—N4—C41—C42	164.0 (2)	B1—C160—C161—C162	−176.8 (2)
N4—C41—C42—C43	54.2 (4)	C160—C161—C162—C163	−0.4 (4)
C41—C42—C43—C44	−54.5 (4)	C161—C162—C163—C164	−0.4 (5)
C42—C43—C44—C45	54.9 (4)	C162—C163—C164—C165	0.5 (5)
C41—N4—C45—C44	54.0 (3)	C163—C164—C165—C160	0.1 (5)
Co1—N4—C45—C44	−162.08 (19)	C161—C160—C165—C164	−0.8 (4)
C43—C44—C45—N4	−54.8 (3)	B1—C160—C165—C164	176.9 (3)
O61—Co2—N7—C510	147.97 (16)	C250—B2—C230—C235	−14.2 (3)
N61—Co2—N7—C510	−125.53 (17)	C240—B2—C230—C235	107.8 (2)
N51—Co2—N7—C510	−32.34 (17)	C260—B2—C230—C235	−132.5 (2)
N8—Co2—N7—C510	63.75 (17)	C250—B2—C230—C231	167.79 (19)
O61—Co2—N7—C610	−88.62 (15)	C240—B2—C230—C231	−70.2 (3)
N61—Co2—N7—C610	−2.11 (15)	C260—B2—C230—C231	49.5 (3)
N51—Co2—N7—C610	91.07 (16)	C235—C230—C231—C232	−1.7 (3)
N8—Co2—N7—C610	−172.83 (15)	B2—C230—C231—C232	176.5 (2)
O51—Co2—N8—C81	78.03 (19)	C230—C231—C232—C233	0.6 (4)
O61—Co2—N8—C81	166.63 (19)	C231—C232—C233—C234	0.7 (4)
N51—Co2—N8—C81	−12.40 (19)	C232—C233—C234—C235	−0.8 (4)
N7—Co2—N8—C81	−97.40 (19)	C233—C234—C235—C230	−0.4 (4)
O51—Co2—N8—C85	−140.36 (17)	C231—C230—C235—C234	1.6 (3)
O61—Co2—N8—C85	−51.76 (16)	B2—C230—C235—C234	−176.6 (2)
N51—Co2—N8—C85	129.21 (17)	C250—B2—C240—C241	156.6 (2)
N7—Co2—N8—C85	44.20 (17)	C230—B2—C240—C241	36.4 (3)
O51—Co2—N51—C57	24.3 (2)	C260—B2—C240—C241	−84.4 (3)
N61—Co2—N51—C57	−72.3 (2)	C250—B2—C240—C245	−29.1 (3)
N7—Co2—N51—C57	−157.0 (2)	C230—B2—C240—C245	−149.3 (2)
N8—Co2—N51—C57	108.9 (2)	C260—B2—C240—C245	89.9 (3)
O51—Co2—N51—C59	−162.18 (19)	C245—C240—C241—C242	0.4 (4)
N61—Co2—N51—C59	101.23 (19)	B2—C240—C241—C242	175.2 (3)
N7—Co2—N51—C59	16.51 (19)	C240—C241—C242—C243	1.1 (5)
N8—Co2—N51—C59	−77.59 (19)	C241—C242—C243—C244	−1.6 (5)
O51—Co2—N61—C67	49.8 (2)	C242—C243—C244—C245	0.6 (5)
O61—Co2—N61—C67	−37.8 (2)	C243—C244—C245—C240	1.0 (5)
N51—Co2—N61—C67	141.2 (2)	C241—C240—C245—C244	−1.5 (4)
N7—Co2—N61—C67	−134.5 (2)	B2—C240—C245—C244	−176.3 (3)
O51—Co2—N61—C69	−146.86 (16)	C240—B2—C250—C251	−40.4 (3)
O61—Co2—N61—C69	125.48 (16)	C230—B2—C250—C251	81.4 (3)
N51—Co2—N61—C69	−55.49 (17)	C260—B2—C250—C251	−159.3 (2)
N7—Co2—N61—C69	28.76 (16)	C240—B2—C250—C255	145.2 (2)
O51—Co2—O61—C61	−41.09 (16)	C230—B2—C250—C255	−93.0 (2)
N61—Co2—O61—C61	55.56 (16)	C260—B2—C250—C255	26.3 (3)
N7—Co2—O61—C61	140.20 (16)	C255—C250—C251—C252	1.5 (4)
N8—Co2—O61—C61	−125.78 (16)	B2—C250—C251—C252	−173.4 (2)
O61—Co2—O51—C51	139.23 (15)	C252—C253—C254—C255	−0.1 (4)
N61—Co2—O51—C51	52.38 (16)	C254—C253—C252—C251	−0.2 (4)
N51—Co2—O51—C51	−40.44 (16)	C250—C251—C252—C253	−0.6 (4)
N8—Co2—O51—C51	−136.86 (16)	C253—C254—C255—C250	1.1 (4)
Co2—O51—C51—C52	−148.37 (16)	C251—C250—C255—C254	−1.7 (4)
Co2—O51—C51—C56	35.1 (3)	B2—C250—C255—C254	173.1 (2)
O51—C51—C52—C53	−177.1 (2)	C250—B2—C260—C261	84.6 (3)
C56—C51—C52—C53	−0.3 (3)	C240—B2—C260—C261	−36.0 (3)
C51—C52—C53—C54	−0.9 (4)	C230—B2—C260—C261	−157.3 (2)
C52—C53—C54—C55	1.2 (4)	C250—B2—C260—C265	−90.6 (3)
C53—C54—C55—C56	−0.4 (4)	C240—B2—C260—C265	148.8 (2)
C54—C55—C56—C51	−0.8 (4)	C230—B2—C260—C265	27.5 (3)
C54—C55—C56—C57	177.4 (2)	C265—C260—C261—C262	0.2 (4)
O51—C51—C56—C55	177.7 (2)	B2—C260—C261—C262	−175.4 (2)
C52—C51—C56—C55	1.2 (3)	C260—C261—C262—C263	−0.4 (5)
O51—C51—C56—C57	−0.5 (3)	C261—C262—C263—C264	0.7 (5)
C52—C51—C56—C57	−177.0 (2)	C262—C263—C264—C265	−0.9 (5)
C59—N51—C57—C56	−174.0 (2)	C263—C264—C265—C260	0.7 (4)
Co2—N51—C57—C56	−0.8 (3)	C261—C260—C265—C264	−0.3 (4)
C59—N51—C57—C58	5.4 (4)	B2—C260—C265—C264	175.2 (2)

### Hydrogen-bond geometry (Å, °)

**Table:**

*D*—H···*A*	*D*—H	H···*A*	*D*···*A*	*D*—H···*A*
C45—H45B···O21	0.97	2.37	2.932 (3)	116
C85—H85A···O61	0.97	2.33	2.907 (3)	117
C135—H135···O21	0.93	2.58	3.289 (3)	133
C231—H231···O61	0.93	2.54	3.261 (3)	135
C18—H18C···Cg1^i^	0.96	2.98	3.559 (3)	120
C610—H61A···Cg1	0.97	2.94	3.886 (2)	165
C45—H45A···Cg2	0.97	3.00	3.658 (3)	126
C210—H21B···Cg3	0.97	2.98	3.898 (2)	158

Symmetry codes: (i) *x*−1, *y*, *z*.
